# Q&A: H1N1 pandemic influenza - what's new?

**DOI:** 10.1186/1741-7007-8-130

**Published:** 2010-10-11

**Authors:** Stephen J Turner, Peter C Doherty, Anne Kelso

**Affiliations:** 1 Department of Microbiology and Immunology, The University of Melbourne, Parkville, Victoria, 3010, Australia; 2 Department of Immunology, St Jude Childrens Research Hospital, 332 Nth Lauderdale, Memphis, TN 38105, USA; 3 WHO Collaborating Centre for Reference and Research on Influenza, 10 Wreckyn Street, North Melbourne, Victoria, 3051, Australia

## The World Health Organization has announced the end of the (H1N1) influenza A (H1N1) pandemic - what does this mean?

In 2009, the new H1N1 pandemic virus exhibited several features that distinguished it from seasonal influenza: it caused major outbreaks in the northern hemisphere summer and autumn, it quickly dominated over other influenza viruses circulating in humans, and it caused widespread disease because of the lack of significant population immunity, particularly in young people. In 2010, the pandemic virus is behaving more like a seasonal influenza virus in that summer outbreaks have not been seen, it is co-circulating with seasonal A(H3N2) and B viruses, and the intensity of transmission is now lower than in 2009. For these reasons, the World Health Organization (WHO) downgraded its pandemic alert from phase 6 to the post-pandemic phase on 10 August 2010. Fortunately, in contrast to descriptions of the 1918 Spanish influenza pandemic, there has been no apparent change in disease severity over the first 18 months of circulation of this virus.

## Does this mean that the pandemic H1N1 influenza virus is no longer a threat?

**Figure 1:**
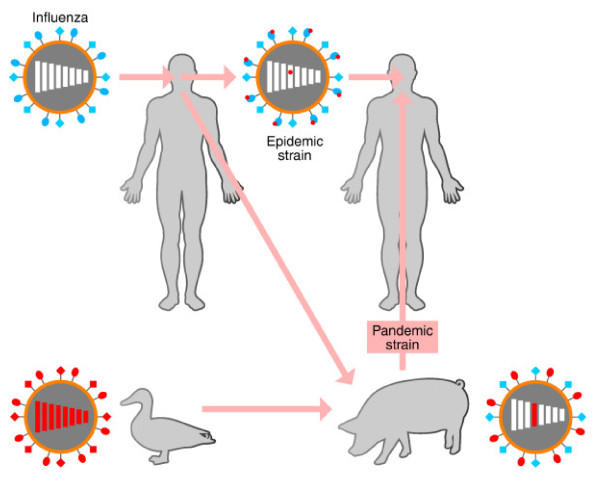
**Mutation and reassortment giving rise to antigenic drift and antigenic shift in different hosts of influenza virus**. The surface hemagglutinin and neuraminidase molecules (blue) of influenza viruses, which play an essential part in viral recognition of and entry into host cells, undergo frequent mutation (antigenic drift) in their human hosts, giving rise to new variants (red dots) that can elude antibodies made in many individuals against the parent virus. Less frequently, entire segments of the eight-segment genome of an avian influenza virus and a human virus become reassorted into the same virion, usually through infection of swine by both viruses, and this can result in a virus that is still adapted to infect humans but expresses an avian hemagglutinin or neuraminidase (antigenic shift) to which there is no prior immunity in human populations. These give rise periodically to pandemics. Figure reproduced with permission from Figure 10-17 of: DeFranco AL, *et al. *2007 [24].

Not necessarily, not altogether. Several features of this virus are a continued cause for concern; for example, most hospitalizations and deaths are still in those under 60 years old. This is probably because people in this age group are less likely to be immune. Furthermore, of those people admitted to hospital in the USA with confirmed influenza (H1N1) 2009 pneumonia, almost two-thirds end up in intensive care. Recent clinical studies have identified risk factors for severe disease that include, but are not limited to, obesity, cardiovascular disease and pregnancy. Importantly, however, about one-third of those who have died with (H1N1) 2009 lacked any known risk factors [1]. It is also of concern that the human influenza (H1N1) 2009 virus can be found in limited instances within pig populations, the species from which it emerged [2]. This increases the opportunity for the virus to reassort with other avian and swine viruses to produce new influenza strains of unpredictable transmissibility and virulence [3]. (Figure 1 illustrates schematically how new pandemic influenza viruses are thought to arise.)

## Is there any sign of reassortment between different viruses?

When we last wrote on this [4], there was no evidence of reassortment between the influenza (H1N1) 2009 virus and other viruses, avian or seasonal. However, the recent re-introduction of influenza (H1N1) 2009 into swine that we have just mentioned does provide the potential, if limited, of reassortment with other swine influenza viruses [3]. Also of concern, Octaviani and colleagues [5] recently used a modified *in vitro *reassortment strategy to ask how easily the current pandemic virus could reassort with a highly pathogenic H5N1 avian influenza, and found, surprisingly, that 85% of the viruses they obtained from this mixing experiment were reassortants. This means that there is excellent genetic compatibility between these two viruses, a characteristic that had been difficult to show between HPAI H5N1 and seasonal influenza viruses current before the pandemic. Reassortant viruses containing the HPAI H5 and N1 components with polymerase subunits from the H1N1 pandemic virus were not only fit but could replicate better than the parent H5N1 virus. This highlights the need for continued surveillance of influenza viruses in the various animal reservoirs, particularly in regions where HPAI H5N1 is endemic.

## What about antigenic drift?

Despite intense surveillance by the WHO Global Influenza Surveillance Network and other systems, significant antigenic drift has not yet been detected in circulating H1N1 2009 viruses. However, we expect that it will appear over the next year or so.

A key driver for antigenic drift in the influenza virus HA glycoprotein is immune pressure by the specific antibody response. Recent serological analyses in a number of countries have found antibodies specific for the pandemic H1N1 virus in up to 40% of surveyed individuals [6], suggesting exposure either by infection or by vaccination. The Centers for Disease Control and Prevention in the USA recently estimated there have been 43 to 88 million cases of pandemic infection [7]. Taken together, these data suggest that a large number of people are immune to the virus. So although it appears that the threshold level of population immunity required to drive antigenic drift has not yet been reached, we might expect antigenic variants to emerge over the next year or so as the pool of susceptible individuals declines.

## Are there any new clues to why susceptibility was so high, especially among younger people, in the first place?

Yes. What has emerged recently is a clear molecular mechanism explaining the lack of immunity to the influenza H1N1 2009 virus in most of the population. Comparison of the hemagglutinin structures of A/California/04/2009 H1N1 and seasonal influenza viruses has shown significant variation within the antigenic sites recognized by specific antibodies [8]. In the same study, a high degree of structural similarity between 2009 H1N1 and 1918 H1N1-like viruses was also evident. Together these analyses provide an explanation for the susceptibility of younger individuals and, conversely, the increased resistance of older individuals who may have been exposed to 1918-like H1N1 viruses in the first half of the 20th century [9].

There are also indications that there may be less cross-protection from T-cell responses to earlier seasonal viruses than had been supposed. Influenza-specific T-cell immunity is often directed against peptide components derived from the more conserved internal viral gene products, such as nucleoprotein, matrix protein 1 or polymerase subunits. T-cell immunity is therefore considered more able to provide heterologous immunity because the targets are more likely to be shared between different influenza strains and subtypes. There are studies that have demonstrated the presence of cross-reactive T cell responses between seasonal and pandemic influenza, supporting the notion that these responses may be important in ameloriating infection in the absence of antibody immunity [10,11]. However, recent data suggest this may not always be the case. Peptides from different influenza strains and commonly targeted by the T-cell response can vary extensively in amino acid sequence, and even when they are able to bind the same major histocompatibility complex (MHC) molecule (on which they are presented for recognition by T cells), T cells specific for one variant peptide may not recognize the other: this has been shown specifically for the seasonal as against the pandemic virus [12]. Thus, despite evidence that many individuals have pre-existing influenza-specific T-cell immunity [13], these findings suggest that, just as with antibody immunity, previous exposure to one subtype does not guarantee effective cross-protective immunity. This may also help to explain why the pandemic virus was able to spread so quickly.

## Do we know any more about why some people are particularly severely affected?

It is clear that in animal models of infection, as well as in human clinical studies, the influenza A (H1N1) virus can replicate more extensively in the lower lung. Clinical data have shown that key risk factors for more severe infection include obesity, diabetes and immunosuppression among other underlying conditions [1]. Clinical studies point to a lack of effective immunity and dysregulated pro-inflammatory responses in those individuals worst affected by infection. For example, a paper presented at the recent Options for the Control of Influenza VII meeting in Hong Kong demonstrated that patients admitted to intensive care had poor immune reactivity (T and B cell) combined with pronounced production of pro-inflammatory cytokines, particularly IL-6. In other unpublished data, at the recent International Congress of Immunology in Japan, Rafi Ahmed presented a molecular characterization of the specific B-cell response in pandemic-infected individuals. By isolating specific B cells and cloning the antibody receptors, he was able to take a census of the types of antibodies induced after infection. Firstly, he showed that about a third of anti-bodies isolated from those individuals who recovered quickly from infection were derived from pre-existing memory B cells and had undergone mutation. This resulted in a repertoire of anti-bodies that were more specific for pandemic than for seasonal influenza. This goes against the 'original antigenic sin' theory, according to which pre-existing immunity to one influenza virus is proposed to limit induction of immunity to subsequent infection with another. Importantly, in one individual admitted to the intensive care unit with severe respiratory distress syndrome induced by pandemic infection, the same analysis of antibody responses demonstrated poor induction of specific antibody. These data together suggest that a combination of underlying risk factors and an inability to mount robust immune responses and to regulate pro-inflammatory responses contributes to disease severity.

## Why is the virus so highly transmissible?

The efficiency of influenza A (H1N1) 2009 virus transmission does not appear to be any greater than that of seasonal influenza. A key factor in the rapid and sustained global spread of the virus during 2009 was the very large pool of susceptible individuals due to low population immunity.

But there have been some recent advances in identifying the molecular determinants of transmission - that is to say, the molecular factors that promote spread of the virus between individuals. Using influenza reverse genetics, two groups introduced known virulence determinants into the influenza A (H1N1) 2009 pandemic virus and used these viruses to study the impact of transmission in a ferret model of infection. Lysine at position 627 of the PB2 protein has been reported to be a virulence determinant in the highly pathogenic HPAI H5N1 avian influenza virus [14] and is absent in the pandemic influenza (H1N1) 2009 viruses. Reassuringly, introduction of this mutation made very little difference to transmission efficiency and pathogenesis and in fact has been reported to attenuate transmission [15].

Another factor is how well the virus binds to receptors in the airways. There is clear evidence that specific amino acids in the hemagglutinin molecule, particularly within the binding site whereby it recognizes its receptor on cells, dictate specificity for either α2,3- or α2,6-linked sialic acids. Human influenza viruses have an aspartic acid (D) at positions 190 and 222 in the hemagglutinin that impart α2,6-sialic acid binding. In contrast, the avian influenza virus preferentially recognizes α2,3-linked sialic acids, and this preference is determined by glutamic acid (E) and glycine (G) at positions 190 and 222, respectively. Of particular interest was an experiment reported by Tumpey and colleagues at the recent Options for the Control of Influenza VII meeting in Hong Kong early in September. They used a mouse-adapted pandemic (H1N1) strain with a D to G mutation at position 222 of hemagglutinin [16]. This was predicted to reduce transmission and pathogenesis in their ferret model of infection. It failed to do either. What was of more interest was that introduction of a I219K mutation into the pandemic virus did result in increased transmission but no change in pathogenesis. This tells us that there is potential for these viruses to undergo further adaptation to human hosts and confirms the need for vigilance in our surveillance.

## How far has the virus become resistant to neuraminidase inhibitors?

Two neuraminidase inhibitors have been widely used in the prophylaxis or treatment of pandemic (H1N1) influenza: oseltamivir (marketed as Tamiflu) and, to a lesser extent, zanamivir (Relenza). Oseltamivir-resistant pandemic strains have been detected, often associated with prolonged treatment of severe cases, but to date there is little evidence of sustained spread of these strains among untreated individuals. As the most common oseltamivir resistance mutation (an H to Y change at position 275) is close to the substrate-binding site of the neuraminidase protein, it was expected from earlier animal studies that such mutants would be less transmissible than their wild-type counterparts. There are conflicting data on this issue. For example, the H275Y oseltamivir resistance mutation emerged in seasonal (H1N1) viruses in late 2007 and spread globally during 2008 in the absence of widespread usage of the drug, suggesting that the mutation had not impaired viral transmissibility. Subsequent work has identified 'permissive' mutations that restored the fitness of these viruses [17]. There are contradicting reports on the impact of oseltamivir resistance on transmission of pandemic influenza strains in ferret models, with one demonstrating lower transmission [18], and the other showing no impact [19]. The reason for this difference is still unclear, so there is plainly a need to monitor the behavior of such drug-resistant viruses in humans carefully.

## How effective has vaccination been?

Initial clinical trials demonstrated that the monovalent influenza (H1N1) 2009 vaccine is immunogenic and capable of inducing levels of antibody that are considered protective [20]. There is also evidence that vaccination reduces not only the risk of infection but also subsequent transmission to others [21]. Vaccination remains the single most effective method of protection from influenza.

That said, it may still be too early to tell just how effective vaccination against the pandemic virus has been. There are two reasons for this. The first is that the initial roll-out of the vaccine occurred too late to affect the first pandemic wave. For example, Australia received the monovalent vaccine in late September 2009. Although this was only 5 months after selection of the vaccine strain, winter was over and the initial pandemic wave had subsided. Importantly, there has been strong collective uptake of the monovalent pandemic vaccine and the later trivalent seasonal vaccine (which included the pandemic (H1N1) strain) in Australia, probably because of an effective public education program. According to recent Australian Government reports, influenza activity is rising even though spring is now beginning in Australia. It will be interesting to see whether the delay in onset of the influenza season and its relatively low activity is due to the extensive vaccination program. We have to wait and see.

## Has there been any progress on making a predictive vaccine or in the mode of flu vaccine manufacture?

The most important impediment to vaccine intervention during the early stages of the pandemic was a delay in availability. Although the full sequence of the new virus was publicly available within days of its identification in April 2009 and a suitable vaccine strain was recommended by WHO just one month later, vaccine production and deployment were significantly delayed by low virus yields in eggs and a number of regulatory hurdles. As a consequence, there is a lot of interest in developing new vaccine strategies that generate more broadly cross-reactive immunity. More recent advances have been in generating antibody responses against conserved regions of the hemagglutinin protein rather than the more variable regions found within the globular head of the protein. In a recent report, Gary Nabel and colleagues demonstrated that a DNA/recombinant adenovirus prime-boost strategy generated antibodies that cross-reacted with antigenically distinct influenza strains [22]. They were able to demonstrate these cross-reactive antibodies target the more conserved stalk region. It is proposed that antibody binding in this region can impede the hemagglutinin conformational changes that are required for virus infectivity. This has been taken a step further by Peter Palese and colleagues [23], who used the hemagglutinin stalk region alone as the immunogen. Again, actively targeting the stalk region in a vaccine strategy induced cross-reactive antibodies, although protective efficacy is yet to be determined. Such strategies are promising but still have a way to go, particularly if pharmaceutical companies are to commit to replacing current vaccine formulations.

## Looking to the future - how quickly would we know if we had a new virus?

Although it took only a few days from initial identification of the influenza (H1N1) 2009 virus in Mexico and California in April to the announcement by WHO that its emergence was a public health event of international concern (indicating its pandemic potential), the virus had in fact been circulating in humans for at least 2 months. Could we have acted earlier to prevent or reduce the impact of the pandemic? More specifically, if we had more active surveillance in the pig population, would this have accelerated detection and response to the new virus in humans? Yes, but not on its own. Early detection of new reassortants in pigs will be useful if it triggers enhanced surveillance for human cases. The most critical need is for rapid detection and laboratory investigation of unusual disease outbreaks combined with open sharing of information and material with national and international health authorities.

## How could we respond if a new pandemic virus emerged in the near future?

Fortunately, our worst fears were not realized in the influenza A (H1N1) 2009 pandemic. However, despite all the advances in technology, surveillance and pandemic planning, the virus spread globally within months, reminding us how difficult it is to control. There were a number of positive outcomes. One was the rapid sharing of information and strains between different parties around the world. This was critical in helping governments and international agencies to shape an appropriate response to an uncertain threat and in enabling manufacturers to produce a new vaccine within 5 months of the first detection of the virus. While some quarters have criticized the response as excessive, it is likely the pandemic would have posed a greater problem in the absence of such interventions. Another positive outcome was the opportunity to evaluate the effectiveness of pandemic plans with a view to ensuring improvements. Furthermore, the emergence of pandemic influenza (H1N1) 2009 stimulated a large amount of research, resulting in new and important knowledge about the virus itself - all important for refining and strengthening our preparedness for future pandemics.
